# Sequence-Based Analysis of Translocations and Inversions in Bread Wheat (*Triticum aestivum* L.)

**DOI:** 10.1371/journal.pone.0079329

**Published:** 2013-11-15

**Authors:** Jian Ma, Jiri Stiller, Paul J. Berkman, Yuming Wei, Jan Rogers, Catherine Feuillet, Jaroslav Dolezel, Klaus F. Mayer, Kellye Eversole, You-Liang Zheng, Chunji Liu

**Affiliations:** 1 Commonwealth Scientific and Industrial Research Organisation (CSIRO) Plant Industry, St Lucia, Brisbane, Queensland, Australia; 2 Triticeae Research Institute, Sichuan Agricultural University, Wenjiang, Chengdu, China; 3 The Genome Analysis Centre and International Wheat Genome Sequencing Consortium, Norwich Research Park, Norwich, United Kingdom; 4 Institute National de Researche Agronomieque (INRA) - University Blaise Pascal, Genetics, Diversity and Ecophysiology of Cereals, Domaine de Crouelle, Clermont Ferrand, France; 5 Centre of the Region Hana for Biotechnological and Agricultural Research, Institute of Experimental Botany, Olomouc-Holice, Czech Republic; 6 Helmholtz Center Munich, German research Center for Environment and Health, Neuherberg, Germany; 7 International Wheat Genome Sequencing Consortium (IWGSC), Bethesda, Maryland, United States of America; 8 School of Plant Biology, The University of Western Australia, Perth, Australia; Kansas State University, United States of America

## Abstract

Structural changes of chromosomes are a primary mechanism of genome rearrangement over the course of evolution and detailed knowledge of such changes in a given species and its close relatives should increase the efficiency and precision of chromosome engineering in crop improvement. We have identified sequences bordering each of the main translocation and inversion breakpoints on chromosomes 4A, 5A and 7B of the modern bread wheat genome. The locations of these breakpoints allow, for the first time, a detailed description of the evolutionary origins of these chromosomes at the gene level. Results from this study also demonstrate that, although the strategy of exploiting sorted chromosome arms has dramatically simplified the efforts of wheat genome sequencing, simultaneous analysis of sequences from homoeologous and non-homoeologous chromosomes is essential in understanding the origins of DNA sequences in polyploid species.

## Introduction

Scientists have long understood that chromosome translocation is a major driving force in shaping genomes during evolution [1]–[5]. It has been well documented that translocations are frequently associated with genomic disorders [6]–[8] and that translocated genes undergo an elevated rate of evolution [1], [9]. Some studies also claim that translocation could alter levels of recombination [10], [11] which is not only a major source of intra-specific variation but also an important constraint in crop improvement programs. Such programs aim to bring together multiple chromosomal segments containing favourable alleles into single plant lines.

The presence of the non-homoeologous translocations between chromosomes 4A, 5A and 7B in the hexaploid wheat (*Triticum aestivum* L., 2n = 6x = 42, genomes AABBDD) is well known, with the first of these translocations was from studies of chromosome pairing and gene-based marker locations [12]. Detailed linkage analyses with molecular markers confirmed the presence of these translocations and also allowed the development of hypotheses on the possible evolutionary origins of these translocation and inversion events [13]–[15]. Analyses of bin-mapped expressed sequence tags (ESTs) showed that, in addition to the two well-known reciprocal translocations and two inversions, a third inversion was also likely involved in generating the structure of the modern chromosome arm ‘4AL’ [16]. It is believed that the 4AL/5AL translocation occurred at the diploid level as it is also present in *T. monococcum* (2n = 2x = 14, genome AA) and the 4A/7B translocation must have occurred at the tetraploid level as it is also present in *T. durum* (2n = 4x = 28, genomes AABB) [14]. Molecular marker profiles of chromosome addition and substitution lines indicated that a 4L/5L translocation may also exist in several other species within the tribe Triticeae [17]. However, due to the limited resolution of marker- or deletion bin-based analyses, fine details for any of these translocations and inversions are still not clear. As none of the techniques currently available allow rapid and accurate detection of these translocations in a given genotype, we still do not know the status of these translocations across the full spectrum of bread wheat and its close relatives. There is also no report on possible contributions of these translocations to wheat speciation.

Recent developments in genome sequencing offer an excellent opportunity to characterize these translocations at the gene level. Synteny-based comparisons of sequences between the sorted wheat chromosomes with those of other grass species identified five distinct segments forming the modern chromosome 4A and putative genes anchoring each of the breakpoints [18]. Similar approaches were used in identifying genes bordering the 7BS/5AL breakpoint on the modern 7BS [19]. Compared with those from previous data which are predominantly based on chromosome pairing or molecular marker analyses, the resolutions offered by these gene-based studies should be significantly higher. However, it is well known that duplications of genes or chromosome segments are common in wheat [20], [21]. Thus, accurate identification of translocation breakpoints could be difficult when analyses are focused on a single chromosome or even a set of homoeologous chromosomes. Further, attempts to trace the evolutionary origins of the modern chromosomes by exploiting genome sequencing data have not been made. Working toward a better understanding of the biological consequences of translocations and tracing the evolutionary origins of the modern chromosomes, we systematically analysed the structures of the 4A, 5A and 7B chromosomes by comparing *Brachypodium* genes against survey sequences of sorted wheat chromosome arms and validating locations of selected genes using bin-mapped ESTs. These analyses identified genes neighbouring breakpoints of these translocation and inversion events thus allowing, for the first time, detailed descriptions of the origins of the modern chromosomes 4A, 5A and 7B of bread wheat at the gene level.

## Materials and Methods

Previous evidence shows that genome segments are highly conserved between wheat and *Brachypodium* although small disruptions of colinearity are not uncommon [18], [22], [23]. Analyses carried out in this study focused on those chromosomal rearrangements evidenced by two or more genes with the same pattern of chromosome arm locations. The known structures of chromosomes 4A, 5A and 7B reported previously [13]–[16], [24] were used to group *Brachypodium* orthologs examined in the initial analyses. Data based on comparison of *Brachypodium* genes with deletion bin-mapped wheat ESTs were used to determine the relative positions and orientations of orthologs within segments of chromosome arms.

As a consequence of the high degree of rearrangement on what is considered the modern chromosome 4A, its arm ratio has been reversed [13]–[16], [24]. As a result, discussion of the various historical states of this chromosome can become difficult to understand. To alleviate this confusion, this manuscript uses 4AS and 4AL to refer to the arms of the original ancestral version of this chromosome, while the modern chromosome arms are referred to as ‘4AS’ and ‘4AL’.

Gene-coding sequences labelled as CDS from *Brachypodium* genome version 192 were downloaded from http://www.plantgdb.org/BdGDB. The locations of orthologs to these *Brachypodium* genes within the survey sequence data of wheat chromosome arms from the genotype ‘Chinese Spring’ were determined using the BLAST++ facility of the International Wheat Genome Sequencing Consortium (IWGSC) (http://www.wheatgenome.org/) hosted by URGI (http://urgi.versailles.inra.fr). The BLASTN algorithm was applied for all analyses using an E value cut-off of 0.0001. Wheat ESTs for individual deletion bins on chromosomes 4A, 5A and 7B were downloaded from http://wheat.pw.usda.gov/GG2/index.shtml. Comparison of bin-mapped ESTs against *Brachypodium* CDS sequences was performed using the BLAST++ BLASTN algorithm with an E value cut-off of 0.00001.

## Results

### Chromosomal Locations of Genes on the Modern 5AL

*Brachypodium* orthologs on this chromosome arm could be placed into two sets. Genes in Set 1 had homoeologous sequences on 5AL, 5BL and 5DL, respectively. The pattern of these chromosome arm locations shows that they are from the original 5AL and were not involved in any interchromosomal translocations. Genes in this set were orthologous to genes on two *Brachypodium* chromosomes, 1 and 4 with *Bradi1g03330* bordering the breakpoint (Tables 1 and S1).

**Table 1:** *Brachypodium* orthologs bordering translocation and inversion breakpoints on chromosome arms 5AL, 7BS, ‘4AL’ and ‘4AS’.

Chromosome arm	Segments#			Border genes##
5AL	5AL	*BP*	4AL	< Bradi1g03330 *BP* Bradi1g75560>
7BS	5AL	*BP*	7BS	> Bradi1g00580 *BP* Bradi1g49340<
‘4AL’	4AL-4	*BP*	5AL	> Bradi1g75530 *BP* Bradi1g03320<
‘4AL’	5AL	*BP*	7BS-1	< Bradi1g00587 *BP* Bradi1g49360>
‘4AL’	4AL-3	*BP*	4AS-2	< Bradi1g74940 *BP* Bradi1g09250>
‘4AL’	4AS-2	*BP*	4AL-2	> Bradi4g14247 *BP* Bradi4g14140>
‘4AS’	4AS-1	*BP*	4AL-1	< Bradi4g14490 *BP* Bradi4g14040<

# Definitions of these chromosome segments are provided in Figure 1. Breakpoints are represented by ‘*BP*’.

## Orientations of genes bordering each of the breakpoints are indicated by ‘>’ (increasing gene IDs) or ‘<’ (decreasing gene IDs). Thus ‘<Bradi1g03330 *BP*’ indicates that ID numbers for genes moving away from the breakpoint increase, and ‘>Bradi1g00580 *BP*’ indicates that ID numbers for genes moving away from the breakpoint decrease.

Genes in Set 2 detected homoeologous sequences on 5AL, 4BL and 4DL, respectively. The pattern of these chromosome arm locations shows that these genes were derived from the original 4AL. Genes in this set were orthologous to genes on three *Brachypodium* chromosomes,1, 4 and 5 with *Bradi1g75560* bordering the breakpoint (Tables 1 and S1).

### Chromosomal Locations of Genes on the Modern 7BS

*Brachypodium* orthologs on this chromosome arm were placed into two sets. Many of the genes in Set 1 detected homoeologous sequences on 7BS, 5BL and 5DL, respectively. The pattern of these chromosome arm locations shows that these genes were derived from the original 5AL. Genes in this set were orthologous to those on *Brachypodium* chromosome 1 with *Bradi1g00580* as the most likely gene bordering the breakpoint (Tables 1 and S2).

Most of the genes in Set 2 detected homoeologous sequences on 7AS, 7BS and 7DS, respectively. The pattern of these chromosome arm locations shows that they were not involved in any interchromosomal translocations. Genes in this set were orthologous to genes on two *Brachypodium* chromosomes, 1 and 3. *Bradi1g49340* can be conservatively assigned as the one bordering the breakpoint (Tables 1 and S2).

### Chromosomal Locations of Genes on the Modern ‘4AL’

*Brachypodium* orthologs on the modern chromosome arm ‘4AL’ could be placed into four sets based on chromosomal locations of wheat sequences they detect. Genes in Set 1 detected sequences on ‘4AL’, 7AS and 7DS, respectively. This pattern of the chromosome arm locations shows that they were derived from the original 7BS. Genes in this set have orthologs on *Brachypodium* chromosomes 1 and 3. These genes could be further placed into two sub-sets based on deletion bin-mapped ESTs (Table S3) but the orientation of genes within these two sub-sets could not be determined.

Genes in Set 2 detected sequences on ‘4AL’, 4BL and 4DL, respectively. This pattern of chromosome arm locations shows that they were derived from the original 4AL. Genes in this set have orthologs on three *Brachypodium* chromosomes, 1, 2 and 4. These genes were placed into three sub-sets based on deletion bin-mapped ESTs (Table S3).

Genes in Set 3 detected sequences on ‘4AL’, 5BL and 5DL, respectively. This pattern of the chromosome arm locations shows that they were derived from the original 5AL. Genes in this set have orthologs on *Brachypodium* chromosome 1. The segment containing these genes is likely flanked by *Bradi1g00587*and *Bradi1g03320* and its orientation is such that the gene IDs, from the centromere, increase (Table S3).

Genes in Set 4 detected homoeologous sequences on ‘4AL’, 4BS and 4DS, respectively. This pattern of the chromosome arm locations shows that they belong to the original 4AS. Genes in this set have orthologs on two *Brachypodium* chromosomes, 1 and 4. The segment containing these genes is likely flanked by *Bradi1g09250* and *Bradi4g14247* (Table S3).

### Chromosomal Locations of Genes on the Modern ‘4AS’

*Brachypodium* orthologs on this chromosome arm were placed into two sets based on the chromosome arm locations of sequences they detect. Many of the genes in Set 1 detected homoeologous sequences on ‘4AS’, 4BL and 4DL, respectively. This pattern of the chromosome arm locations shows that these genes belonged to the original 4AL. Genes in this set have orthologs on two *Brachypodium* chromosomes, 1 and 4. The chromosome segment containing these genes is likely flanked by *Bradi1g74922* and *Badi4g14040* (Table S4).

Five genes were found to likely belong to Set 2 on this chromosome arm and they have orthologs on *Brachypodium* chromosome 4. These genes detect homoeologous sequences on ‘4AS’, 4BS and 4DS, respectively. This pattern of the chromosome arm locations shows that they were translocated from the original 4AS to the modern ‘4AS’. Two of them (*Bradi4g14830* and *Bradi4g14990*) also detected sequences on ‘4AL’, showing that they are duplicated on this chromosome. The reason for their inclusion in this set is that genes flanking them all detected sequences on the three short arms of the homoeologous group 4 chromosomes (Table S4).

## Discussion

**Figure 1:**
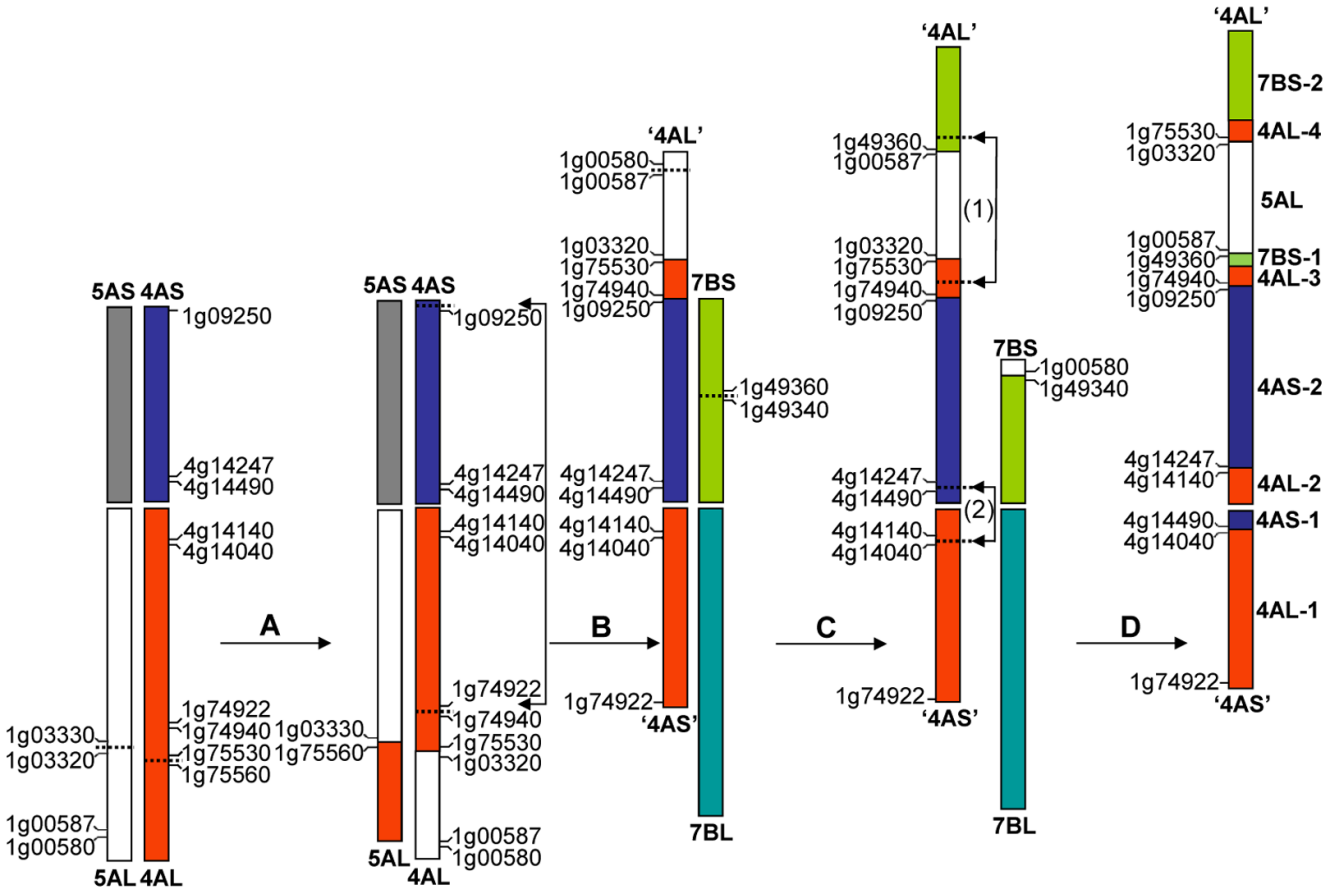
The structures of modern chromosomes 4A, 5A and 7B and their evolutionary origins. The translocation and inversion events resulted in these structures are marked as A: 4AL/5AL reciprocal translocation; B: 4A pericentric inversion; C: ‘4AL’/7BS reciprocal translocation, and D: one paracentric (1) and one pericentric (2) inversion on chromosome 4A. Loci defining these translocation and inversion breakpoints and the ancestral forms of the nine segments on the modern chromosome 4A are indicated.

By analysing *Brachypodium* genes against survey sequences of sorted wheat chromosome arms and by analysing *Brachypodium* genes against deletion bin-mapped wheat ESTs, we have identified *Brachypodium* orthologs bordering several translocation and inversion breakpoints on the modern wheat chromosomes 4A, 5A and 7B. This new analysis allowed detailed description of the evolutionary origins of these bread wheat chromosomes at the gene level (Fig. 1).

Several ESTs were mapped to each of the three smallest segments on the modern chromosome arm ‘4AL’ (4AL-3, 4AL-4 and 7BS-1, respectively) [16]. However, we found corresponding *Brachypodium* genes for only a few of these ESTs (Table S3) preventing us from accurate allocation and orientation of the *Brachypodium* orthologs on these segments. Considering the orders of the genes on the original chromosomes and the translocation and inversion events, orientations of these fragments could be deduced as (from centromere): increasing gene IDs for those in 4AL-3, decreasing gene IDs for those in 4AL-4 and decreasing gene IDs for those in 7BS-1 (Fig. 1; Tables S2 and S3). The deduced orientations for those genes on 4AL-3 and 4AL-4 seem to be in agreement with the findings by Hernandez et al. [18] who reported that genes bordering the ‘segment C’ derived from the original 4AL on the modern ‘4AL’ have opposite orientations. Our data confirm that these genes form two separate 4AL segments (4AL-3 and 4AL-4 in Fig. 1) on the modern ‘4AL’ as proposed by Miftahudin et al. [16].

Previous models of the structures of chromosomes 5A and 7B are highly consistent [12]–[14]. The structure of the chromosome 4A is less clear. Based on next-generation sequencing Hernandez et al. [18] suggested that five segments form the modern chromosome 4A. Our results show that this chromosome contains at least nine segments (Fig. 1), a structure similar to that deducted from deletion bin-mapped ESTs [16]. However, we found that many of the deletion-bin mapped ESTs detect sequences on large numbers of chromosome arms (Table S5) thus could not be used reliably in tracing the origins of a gene or a chromosome segment. We also found evidence showing that a small segment (4AS-1 in Fig. 1) relocated from the original 4AS to the modern ‘4AS’ during the second pericentric inversion (Fig. 1).

Analysis of chromosome pairing showed that the terminal segment of the modern ‘4AS’ is homoeologous to 4BS and 4DS [12], indicating the first pericentric inversion (marked as event ‘B’ in Fig. 1) was proximal to the telomere of the original 4AS. This chromosome pairing result seems to be supported by the locations of two deletion bin-mapped ESTs, BE518074 and BE494743 [16]. These two ESTs, when analysed by sequence similarity against sequences of the sorted wheat chromosome arms showed that, although both detected sequences on the modern chromosome arm ‘4AS’, neither gave a clear matching chromosome pattern to confirm that they were translocated from the original 4AS to the modern ‘4AS’ (Table S5). BE518074 matched *Brachypodium* gene *Bradi2g54210.* However *Brachypodium* orthologs on either side of this gene failed to detect sequences on each of the three short arms of the homoeologous group 4 chromosomes (Table S6). Thus the question whether the inversion breakpoint was proximal to the telomere of the original 4AS remains unanswered.

The strategy of sequencing the wheat genome based on sorted chromosomes or chromosome arms has significantly simplified wheat genome research as it circumvents many of the complications caused by the hexaploid nature of this species [18], [19], [25], [26]. However, caution is required when using results obtained from such a strategy to trace the evolutionary origin of a given gene or a chromosome segment. For example, *Bradi1g00227* and *Bradi1g02980* were reported to flank the original 5AL segment on the modern ‘4AL’ [18]. Our results showed that the segment flanked by *Brad1g00450* and *Bradi1g00580* was actually translocated from the original 5AL to the modern 7BS as the majority of the genes residing on this segment have homoeologous sequences on 7BS, 5BL and 5DL (Fig. 1, Table S7). The location of these genes on 7BS is further supported by the fact that most of these genes were found to be present on the 7BS syntenic build [19]. Many of the genes between *Bradi1g00227* and *Bradi1g00460* detected homoeologous sequences on the modern ‘4AL’. However, most of them also detected multiple sequences on chromosomes belonging to several homoeologous groups. For example, *Bradi1g00227* detects homoeologous sequences on 21 chromosome arms belonging to six of the seven homoeologous groups of bread wheat (Table S7). The multiple locations of many genes in bread wheat are not surprising considering its hexaploid nature and the well-known fact that duplications of genes or chromosome segments are common in this species [20], [21].

Another example is the 7BS syntenic build where *Bradi1g49497* was suggested to be one of the genes neighbouring the 7BS/5AL breakpoint on this chromosome arm [19]. We found that the anchoring gene for this breakpoint is *Bradi1g49340. Bradi1g49497* detected sequences on both 7BS and 4AL and most of the *Brachypodium* orthologs between these two genes belong to a segment translocated from the original 7BS to the modern ‘4AL’ as they detected homoeologous sequences on ‘4AL’, 7AS and 7DS, respectively (Table S8). These examples demonstrate that a more in-depth simultaneous analysis of sequences from homoeologous and non-homoeologous chromosomes is essential in understanding the origins of a DNA sequence in polyploid species.

## Supporting Information

Table S1 ***Brachypodium*** **orthologs on modern chromosome arm 5AL.**
(XLSX)

Table S2 ***Brachypodium*** **orthologs on modern chromosome arm 7BS.**
(XLSX)

Table S3 **Brachypodium orthologs on modern chromosome arm ‘4AL’.**
(XLSX)

Table S4 ***Brachypodium*** **orthologs on modern chromosome arm ‘4AS’.**
(XLSX)

Table S5 **Chromosome arm locations of wheat sequences detected by ESTs reported by Miftahudin et al** [**16**] **.**
(XLSX)

Table S6 **Wheat sequences detected by** ***Brachypodium*** **orthologs on either side of** ***Bradi2g54210.***
(XLSX)

Table S7 **Chromosome arm locations of wheat sequences detected by** ***Brachypodium*** **genes between** ***Bradi1g00227*** **and** ***Bradi1g00620.***
(XLSX)

Table S8 ***Brachypodium*** **orthologs on the modern chromosome arm ‘4AL’ translocated from the original 7BS.**
(XLSX)

## References

[1] Burt DW, Bruley C, Dunn IC, Jones CT, Ramage A, et al (1999) The dynamics of chromosome evolution in birds and mammals. Nature 402: 411–412. 10586880 10.1038/46555

[2] Sankoff D, Nadeau JH (2003) Chromosome rearrangements in evolution: From gene order to genome sequence and back. Proceedings of the National Academy of Sciences 100: 11188–11189. 10.1073/pnas.2035002100 PMC208729 14506293

[3] Colson I, Delneri D, Oliver SG (2004) Effects of reciprocal chromosomal translocations on the fitness of Saccharomyces cerevisiae. EMBO reports 5: 392–398. 15105830 10.1038/sj.embor.7400123 PMC1299034

[4] Brown JD, O’Neill RJ (2010) Chromosomes, conflict, and epigenetics: chromosomal speciation revisited. Annual review of genomics and human genetics 11: 291–316. 10.1146/annurev-genom-082509-141554 20438362

[5] Morrow JD, Cooper VS (2012) Evolutionary effects of translocations in bacterial genomes. Genome Biology and Evolution 4: 1256–1262. 23160175 10.1093/gbe/evs099 PMC3542574

[6] Lupski JR (1998) Genomic disorders: structural features of the genome can lead to DNA rearrangements and human disease traits. Trends in genetics 14: 417. 9820031 10.1016/s0168-9525(98)01555-8

[7] Kuppers R, Dalla-Favera R (2001) Mechanisms of chromosomal translocations in B cell lymphomas. Oncogene 20: 5580–5594. 11607811 10.1038/sj.onc.1204640

[8] Rowley JD (2004) A new consistent chromosomal adnormality in chronic myelogeneus leukaemia identified by quinacrine fluorescence and Giemsa staining. Landmarks in Medical Genetics: Classic Papers with Commentaries 243: 104. 10.1038/243290a0 4126434

[9] Hao W, Golding GB (2009) Does gene translocation accelerate the evolution of laterally transferred genes? Genetics 182: 1365–1375. 19474197 10.1534/genetics.109.104216 PMC2728873

[10] McKim KS, Howell AM, Rose AM (1988) The effects of translocations on recombination frequency in Caenorhabditis elegans. Genetics 120: 987–1001. 3224815 10.1093/genetics/120.4.987 PMC1203590

[11] Sherizen D, Jang JK, Bhagat R, Kato N, McKim KS (2005) Meiotic recombination in Drosophila females depends on chromosome continuity between genetically defined boundaries. Genetics 169: 767–781. 15545646 10.1534/genetics.104.035824 PMC1449117

[12] Naranjo T, Roca A, Goicoechea P, Giraldez R (1987) Arm homoeology of wheat and rye chromosomes. Genome 29: 873–882.

[13] Liu C, Atkinson M, Chinoy C, Devos K, Gale M (1992) Nonhomoeologous translocations between group 4, 5 and 7 chromosomes within wheat and rye. Theoretical and Applied Genetics 83: 305–312. 24202512 10.1007/BF00224276

[14] Devos K, Dubcovsky J, Dvořák J, Chinoy C, Gale M (1995) Structural evolution of wheat chromosomes 4A, 5A, and 7B and its impact on recombination. Theoretical and Applied Genetics 91: 282–288. 24169776 10.1007/BF00220890

[15] Nelson JC, Sorrells ME, Van-Deynze A, Lu YH, Atkinson M, et al (1995) Molecular mapping of wheat: major genes and rearrangements in homoeologous groups 4, 5, and 7. Genetics 141: 721. 8647405 10.1093/genetics/141.2.721 PMC1206768

[16] Miftahudin., Ross K, Ma XF, Mahmoud A, Layton J, et al (2004) Analysis of expressed sequence tag loci on wheat chromosome group 4. Genetics 168: 651–663. 15514042 10.1534/genetics.104.034827 PMC1448824

[17] King I, Purdie K, Liu C, Reader S, Pittaway T, et al (1994) Detection of interchromosomal translocations within the Triticeae by RFLP analysis. Genome 37: 882–887. 18470131 10.1139/g94-125

[18] Hernandez P, Martis M, Dorado G, Pfeifer M, Gálvez S, et al (2012) Next-generation sequencing and syntenic integration of flow-sorted arms of wheat chromosome 4A exposes the chromosome structure and gene content. The Plant Journal 69: 377–386. 21974774 10.1111/j.1365-313X.2011.04808.x

[19] Berkman PJ, Skarshewski A, Manoli S, Lorenc MT, Stiller J, et al (2012) Sequencing wheat chromosome arm 7BS delimits the 7BS/4AL translocation and reveals homoeologous gene conservation. Theoretical and Applied Genetics 124: 423–432. 22001910 10.1007/s00122-011-1717-2

[20] Salse J, Bolot S, Throude M, Jouffe V, Piegu B, et al (2008) Identification and characterization of shared duplications between rice and wheat provide new insight into grass genome evolution. The Plant Cell Online 20: 11–24. 10.1105/tpc.107.056309 PMC2254919 18178768

[21] Brenchley R, Spannagl M, Pfeifer M, Barker GLA, D’Amore R, et al (2012) Analysis of the bread wheat genome using whole-genome shotgun sequencing. Nature 491: 705–710. 23192148 10.1038/nature11650 PMC3510651

[22] Griffiths S, Sharp R, Foote TN, Bertin I, Wanous M, et al (2006) Molecular characterization of Ph1 as a major chromosome pairing locus in polyploid wheat. Nature 439: 749–752. 16467840 10.1038/nature04434

[23] Vogel JP, Garvin DF, Mockler TC, Schmutz J, Rokhsar D, et al (2010) Genome sequencing and analysis of the model grass Brachypodium distachyon. Nature 463: 763–768. 20148030 10.1038/nature08747

[24] Mickelson-Young L, Endo T, Gill B (1995) A cytogenetic ladder-map of the wheat homoeologous group-4 chromosomes. Theoretical and Applied Genetics 90: 1007–1011. 24173055 10.1007/BF00222914

[25] Berkman PJ, Skarshewski A, Lorenc MT, Lai K, Duran C, et al (2011) Sequencing and assembly of low copy and genic regions of isolated Triticum aestivum chromosome arm 7DS. Plant Biotechnology Journal 9: 768–775. 21356002 10.1111/j.1467-7652.2010.00587.x

[26] Doležel J, Kubaláková M, Paux E, Bartoš J, Feuillet C (2007) Chromosome-based genomics in the cereals. Chromosome Research 15: 51–66. 17295126 10.1007/s10577-006-1106-x

